# Allometry of the quasi-pipe (qPipe) model for estimating tree leaf area and tree leaf mass applied to plant functional types

**DOI:** 10.1038/s41598-023-37112-1

**Published:** 2023-06-19

**Authors:** Akihiro Sumida, Yoshiyuki Inagaki, Takuya Kajimoto, Masumi Katsuno-Miyaura, Akira Komiyama, Nahoko Kurachi, Tomiyasu Miyaura, Shigeaki F. Hasegawa, Toshihiko Hara, Kiyomi Ono, Masahito Yamada

**Affiliations:** 1 Graduate School of Life and Environmental Sciences, Kyoto Prefectural University, 1-5 Shimogamohangi, Sakyo-Ku, Kyoto, 606-8522 Japan; 2 Shikoku Research Center, Forestry and Forest Products Research Institute, 2-915 Asakuranishi, Kochi, 780-8077 Japan; 3 Sado Island Center for Ecological Sustainability, Niigata University, 94-2 Koda, Sado, Niigata 952-2206 Japan; 4 Hiraoka Forest Research Institute, Aoyama, Otsu, Shiga 520-2101 Japan; 5 Gifu University, 1-1 Yanagido, Gifu, 501-1193 Japan; 6 Faculty of Advanced Science and Technology, Ryukoku University, Seta Oe-Cho, Otsu, Shiga 520-2194 Japan; 7 Faculty of Human Sciences and Cultural Studies, Yamanashi Eiwa College, 888 Yokonemachi, Kofu, Yamanashi 400-8555 Japan; 8 Institute of Low Temperature Science, Hokkaido University, N19W8, Sapporo, 060-0819 Japan

**Keywords:** Ecology, Forest ecology, Plant ecology, Forestry, Ecological modelling

## Abstract

The allometry of the pipe model quantifies the approximate proportionality between the tree leaf amount and the stem cross-sectional area at the crown base (*A*_CB_). It is useful for estimating and modeling carbon fixation abilities of trees but requires climbing the tree and is thus unsuitable for large-scale studies. Here, we adopted a previously proposed allometry (hereafter the quasi-pipe (qPipe) model allometry) formulating the relationship between the tree leaf amount and a surrogate of *A*_CB_, *A*_CB_Est_, calculated from tree dimensions measurable from the ground. Using published/unpublished data for 962 trees of 159 species collected between tropical rainforests and boreal forests, we established pipe and qPipe model allometries for evergreen-conifer, deciduous-conifer, evergreen-broadleaf, and deciduous-broadleaf plant functional types (PFTs). For the leaf area per tree (LA), allometric lines on a log–log plane were almost identical among the four PFTs in both models, with slopes of ~ 1. For the leaf mass per tree (LM), however, the allometric lines separated among the four PFTs in both models and had slopes greater than 1, indicating that the proportionality assumed in the pipe model held for LA but not LM. The applicability of the qPipe model in estimating the stand-scale leaf amount was further examined.

## Introduction

The estimation of the amount of leaves in forests at a global scale is essential in projecting and modeling carbon fixation abilities on Earth^1^. Remote-sensing technologies, such as satellite observations and light detection and ranging, allow the estimation of the amount of leaves per unit land area, such as the estimation of the leaf area index (LAI, the leaf area per unit land area), over a wide area^2^. Still, methods of obtaining the ground truth of the LAI via fieldwork-based stand-scale ecological studies are needed to support the remote-sensing technologies^3,4^. One of the major ground-truth methods is the allometric method^5^, in which the amount of leaves per tree is estimated using a known relation between the amount of leaves (leaf area or leaf mass) and measures of the tree size such as the trunk diameter at breast height (DBH) and tree height. The LAI of a stand is estimated by summing the allometrically estimated tree leaf area for all trees of a stand and dividing by the stand area. One advantage of the allometric method is that it allows us to calculate the confidence intervals and/or prediction intervals of the total amount of leaves per stand, as these intervals can be calculated from estimates of the prediction intervals of each individual tree via regression calculation^6^ and the rule of propagation of errors^7,8^.

However, there are problems in implementing the allometric method. First, when the DBH or the combination of the DBH and tree height are used as the predictor variable(s) of the tree leaf amount, the allometric equation prepared for a stand is in general not applicable to the same stand in the future or to other stands. A typical reason is that the leaf amount of a given tree may not change or even decrease, instead of increasing, despite the DBH and tree height continuing to increase with age^8,9^. This is one reason why both the intercept and slope coefficients of a linear allometric equation change with stand age^10–12^.

The allometric equation known as the pipe model of the tree shape^13,14^ is suitable for estimating the leaf amount of a tree. The model predicts the tree leaf amount as being approximately directly proportional to the stem cross-sectional area at the crown base (*A*_CB_) or the crown base stem diameter squared (*D*_CB_^2^). As the pipe model uses *A*_CB_ or *D*_CB_^2^ as the predictor of the leaf amount, it can express a constant or decreasing leaf amount over time; a tree with a decreasing leaf amount generally has a decreasing crown length because the speed of the rise of crown base location exceeds the height growth owing to the death of lower branches^8,9^. Such a rapid crown-base rise often leads to a decrease in *A*_CB_ and the leaf amount^8,9^.

After the publication of the pipe model, many studies confirmed the validity of the model and related it to the relationship between the tree leaf amount and stem sapwood area at the crown base^15,16^. In this paper, however, we refer to the pipe model as the approximate proportional relationship of the tree leaf amount with the stem cross-sectional area at the crown base *A*_CB_ or *D*_CB_^2^ and not the relationship with the sapwood area of a trunk as in other studies. The validity of the pipe model implies that the ‘pipe model ratio’^17^ (defined as the leaf amount per unit area of the stem cross section or unit sapwood area) is constant irrespective of the tree size. Meanwhile, little attention has been paid to whether the amount of leaves in the pipe model should be expressed on a leaf mass basis or a leaf area basis^16^. The leaf amount in the original pipe model^13^ was expressed on a leaf mass basis, whereas some later studies expressed the leaf amount on a leaf area basis. If the pipe model ratio is constant for both the tree leaf area and tree leaf mass, the tree leaf mass per tree leaf area (tree LMA) must be constant between trees of different size in a stand. However, studies of an individual-leaf scale suggest that the LMA of an individual leaf is affected by the light conditions such that it decreases with decreasing light from the canopy surface downward^18–21^. It is thus expected that the tree LMA is smaller for shorter trees in a stand than for taller trees in the same stand. If so, the pipe model ratio being constant between trees cannot hold for both the leaf area and leaf mass simultaneously. In other words, the proportionality between *A*_CB_ or *D*_CB_^2^ and the tree leaf amount cannot hold simultaneously for both the leaf area and leaf mass, unless the tree LMA is constant among trees of different size in a stand. This means that when the allometric line is drawn on a log–log plane, the slope should be different between the allometry for the leaf area and that for the leaf mass.

Care must be taken when estimating the slope of a regression line. In estimating the slope, the use of a regression method called Model II regression is recommended as it calculates reasonable estimates of the slope by considering residuals of both 'x' and 'y' simultaneously^6,22^. Meanwhile, the regression method used for predicting the 'y' value from a given value ‘x’ is called Model I regression. As the slope of the regression line of Model II regression is known to be greater than that of the Model I regression^22^, allometric analyses for establishing the relationship (Model II) and for prediction (Model I) should be conducted simultaneously.

Another practical problem of the pipe model is that one needs to climb to the base of the tree crown to measure *A*_CB_. This issue has partly been solved adopting a method for approximating *A*_CB_ without the need for climbing^12^. The following relationship was obtained from measurements for 156 trees of *Betula ermanii* Cham. in three stands^12^:

1 $$A_{{{\text{CB}}}} /A_{{{\text{BH}}}} = L_{{\text{C}}} /H_{{ > {1}.{3}}} ,$$

where *A*_BH_ is the stem cross-sectional area at a 1.3-m height, *L*_C_ is the crown length (i.e., the difference between the tree height and height of the crown base), and *H*_>1.3_ the length of the stem part above a height of 1.3 m. This is equivalent to assuming that there is a virtual paraboloid of revolution that has its apex at the treetop, and cross-sectional areas at the crown base (i.e., at the length *L*_C_ from the treetop) and at the breast height being *A*_CB_ and *A*_BH_, respectively. It follows from Eq. (1) that *A*_CB_ can be approximated as^12^

2 $$A_{{{\text{CB}}\_{\text{Est}}}} = A_{{{\text{BH}}}} R_{{{\text{CROWN}}}} ,$$

where *A*_CB_Est_ is an estimate of the stem cross-sectional area at the crown base, and *R*_CROWN_ is the crown ratio defined as *L*_C_/*H*_>1.3_. As *A*_BH_, *L*_C_, and *H*_>1.3_ are all measurable from the forest floor, the use of *A*_CB_Est_ as the surrogate of *A*_CB_ is practical in estimating the tree leaf amount just like for the pipe model.

So far, the application of Eq. (2) has only been reported for two species^12,23^. Both studies showed that the actual measured *A*_CB_ and its estimate *A*_CB_Est_ were not statistically different, such that *A*_CB_ and *A*_CB_Est_ were treated as though they were convertible predictors of the leaf amount. The allometry for the tree leaf area using *A*_CB_Est_ as a predictor variable was found to be not statistically different from the pipe model allometry using *A*_CB_ as a predictor variable^12^. When estimating the LAI of the stand, the tree leaf area for trees whose *A*_CB_ could not be directly measured in the cited study was estimated by substituting *A*_CB_Est_ into an existing pipe model allometric equation, namely the *A*_CB_–tree leaf area relationship^12^. In contrast, the present study presumes that the pipe model allometry is unavailable owing to a lack of *A*_CB_ data, and this is why the allometry that uses *A*_CB_Est_ as a predictor variable is needed. Therefore, we deal with the allometry using *A*_CB_Est_ and that using *A*_CB_ (i.e., the pipe model allometry) differently in the present study. Hereafter, the allometry using *A*_CB_Est_ as a predictor variable for the tree leaf amount^12^ is referred to as the quasi-pipe (qPipe) model allometry to indicate the difference. The form of the allometric equations in the present study is given in the caption of Table 1. A comparison between the pipe and qPipe model allometries is presented as equations (S1) and (S5) in Supplementary Information S1 and Supplementary Fig. S1.

**Table 1:** Coefficients of regression lines assuming *Y* = CF × Elevation × *X*^Slope^ in Figs. 1, 2, 3 and 4.

Figure number (regression type)	Response variable	Predictor variable (s)	PFT	Elevation 95% Conf. int	Slope 95% Conf. int	CF	*R*^2^	*n*
1 (SMA)	ln(*A*_CB_Est_)	ln(*A*_CB_), PFT	EC	1.065^a^ 0.970–1.169	1.000 0.980–1.018	–	0.978	225
			DC	1.036^a,b^ 0.889–1.208	0.995 0.967–1.024	–	0.987	57
			EB	0.880^c^ 0.798–0.970	0.990 0.975–1.006	–	0.967	453
			DB	1.046^c,d^ 0.935–1.171	1.014 0.995–1.034	–	0.972	227
2a (SMA)	ln(LA)	ln(*A*_CB_), PFT	EC	5097^a^ 3631–7156	**1.091** 1.029–1.157	–	0.920	107
			DC	8880^b^ 2612–30190	1.146 0.950–1.383	–	0.907	15
			EB	3347 ^a,b^ 2651–4225	1.004 0.967–1.042	–	0.860	445
			DB	3371^a,b^ 2510–4530	1.006 0.956–1.059	–	0.895	201
2b (SMA)	ln(LM)	ln(*A*_CB_), PFT	EC	1058^a^ 869.5–1287	**1.106** 1.067–1.145	–	0.943	221
			DC	732.3^b^ 454.7–1179	**1.197** 1.111–1.289	–	0.933	57
			EB	473.7^c^ 374.2–599.6	**1.060** 1.023–1.098	–	0.865	447
			DB	262.5^d^ 194.9–353.5	**1.061** 1.011–1.113	–	0.882	227
2c (SMA)	ln(LA)	ln(*A*_CB_Est_), PFT	EC	4830^a^ 3385–6892	**1.107** 1.040–1.178	–	0.900	107
			DC	12640^b^ 2771–57620	1.239 0.993–1.545	–	0.878	15
			EB	3915^a,b^ 3058–5012	1.020 0.982–1.061	–	0.848	445
			DB	2959^b,c^ 2178–4020	0.978 0.927–1.032	–	0.891	201
2d (SMA)	ln(LM)	ln(*A*_CB_Est_), PFT	EC	982.2^a^ 788.5–1223	**1.105** 1.062–1.151	–	0.927	221
			DC	880.7^b^ 532.4–1457	**1.253** 1.162–1.352	–	0.932	57
			EB	575.9^c^ 453.3–731.5	**1.082** 1.044–1.120	–	0.864	447
			DB	229.3^d^ 171.5–306.7	1.033 0.985–1.084	–	0.890	227
3a (GLMM)	ln(LA)	ln(*A*_CB_)	Total PFTs	2370	0.940	1.065	–	768
3b (GLMM)	ln(LA)	ln(*A*_CB_Est_)	Total PFTs	2742	0.955	1.077	–	768
4a (GLMM)	ln(LM)	ln(*A*_CB_Est_)	EC	854.4	1.078	1.023	–	221
4b (LS)	ln(LM)	ln(*A*_CB_Est_)	DC	571.4	1.172	1.059	–	57
4c (GLMM)	ln(LM)	ln(*A*_CB_Est_)	EB	293.8	0.973	1.073	–	447
4d (GLMM)	ln(LM)	ln(*A*_CB_Est_)	DB	180.3	1.017	1.073	–	227

See the main text for abbreviations and units. All regressions were significant at *P* < 0.000. Slope values of SMA regressions in boldface indicate a significant difference from 1. Different letters for the estimates of the elevation in SMA regression indicate significant differences via pair-wise comparisons with the Sidak correction. In all GLMM analyses, species was selected as the random effect of elevation.

The reason why Eq. (2) holds for estimating *A*_CB_ is not biologically based but is simply based on a general characteristic that the tree trunk tapers toward the treetop (see the Discussion). As Eq. (2) does not require the determination of regression coefficients such as the intercept and slope, if Eq. (2) is found to hold for other species, the qPipe model would be a convenient tool for estimating the tree leaf amount when no allometric relationship is available for a particular stand.

To test the applicability of the qPipe model to other species at a global scale, plant functional types (PFTs) [i.e., the combination of evergreen/deciduous (E or D) and broadleaved/coniferous (B or C)], would be useful, as PFTs are identifiable through remote sensing and are related to ecosystem-scale physiological functioning^24,25^. Hereafter, the four PFTs are abbreviated as EB, EC, DB, and DC (excluding monocods such as palm-tree species).

In this study, we propose the qPipe model allometry for estimating the stand-scale leaf biomass and LAI in large-scale studies, by pooling the data of the tree species of each PFT. Published/unpublished data for 962 trees collected from tropical rainforests in Southeast Asia, temperate forests of Japan and Tasmania, and boreal forests of Japan and Siberia are used in analyses. The number of trees (species) is 453 (102), 225 (10), 227 (44), and 57 (3) for EB, EC, DB, and DC PFTs, respectively. The list of species and number of trees are given in Supplementary Information S3. Hereafter, the term ‘leaf mass’ refers to the leaf dry mass. The leaf area for broadleaf species is the one-sided area of a leaf. For conifer species, the leaf area in all data used in the analyses is measured as the projected area by detaching all needles from the stem. We first examine (1) whether the relationship between *A*_CB_ and *A*_CB_EST_ is 1:1 using standardized major axis (SMA) regression (the smatr library^22^ in R^26^), which is Model II regression suitable for estimating the slope^6^. Then (2) for the four PFTs, the pipe model allometries are explored and compared with qPipe models through SMA regression. Next, (3) to estimate the leaf area and leaf mass and their 95% prediction intervals using Model I regression, the qPipe models are formulated via general linear mixed models (GLMMs), with species being designated as a random effect. This regression method is a Model I regression^6^. In both SMA regression and GLMMs, best models are selected using the Akaike information criteria (AIC). Lastly, (4) to present an example of how LAI estimates for one stand differ between the pipe and qPipe model allometries, a comparison is made between LAI estimates using published 20-year changes (from 21 to 40 years of stand age) in the LAI measured in an evergreen conifer ‘hinoki’ cypress (*Chamaecyparis obtusa* (Siebold et Zucc.) Endl.) stand^8^. Using tree census data including data of the crown base stem diameter recorded for each tree each year over the 20 years^9^, the LAIs of each year and their 95% prediction intervals are calculated by adopting three methods; i.e., adopting the pipe model allometry prepared for the stand^8^ using the measured *A*_CB_ as the predictor (‘site-specific pipe model’), the qPipe model allometry prepared using *A*_CB_Est_ as the predictor, where *A*_CB_Est_ is calculated using the same tree census data (‘site-specific qPipe model’; see Supplementary Information S1), and the qPipe model allometry of the PFT obtained in (3) using *A*_CB_Est_ (‘global qPipe model’). In this comparison, the 95% prediction intervals of each LAI estimates are calculated through an error propagation of 95% prediction intervals of the leaf area estimates of individual trees^7,8^. All statistical calculations are performed using R^26^.

## Results

### Comparison between measured* A*_CB_ and estimates *A*_CB_Est_

**Figure 1:**
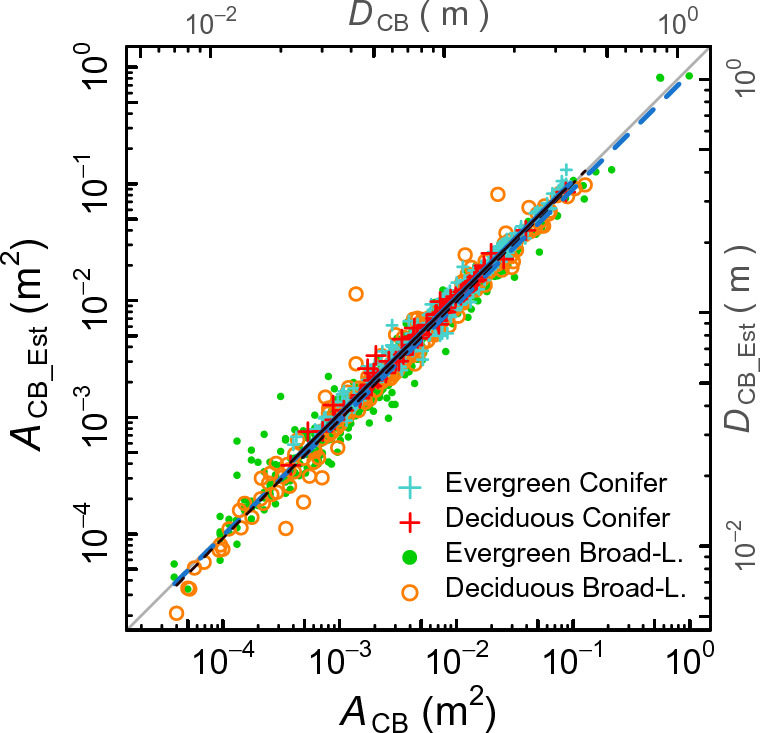
Relationship between *A*_CB_ and *A*_CB_Est_ obtained using SMA regression. PFTs are included as the categorical variable of an SMA regression equation, and their elevation (values of *A*_CB_Est_ at *A*_CB_ = 1 m^2^) and slope are given in Table 1. Regression lines are drawn between horizontal data ranges of respective PFTs, where the blue solid line is for the EC PFT, the black solid line for the DC PFT, blue dashed line for the EB PFT, and black dashed line for the DB PFT. *D*_CB_ and *D*_CB_Est_ indicate scales of diameter converted from *A*_CB_, and *A*_CB_Est_, respectively.

The measured values of *A*_CB_ (m^2^) and the estimates *A*_CB_Est_ (m^2^) calculated with Eq. (2) were compared among PFTs using SMA regression (Fig. 1). Both *A*_CB_ and *A*_CB_Est_ were log-transformed in the regression calculation. The best model was the one having ln(*A*_CB_) and the four PFTs (categorical variable) as the predictor variables (Table 1). The allometric line was drawn for each PFT. Figure 1 shows that the relationship was almost 1:1 for each PFT. The slopes of the four PFTs ranged between 0.99 and 1.01, without significant differences from 1.0 (Table 1). Pair-wise differences were significant between the elevations (intercepts) of the regression lines of the four PFTs except between the elevations of the EC and DC PFTs and between the elevations of the EB and DB PFTs. However, these differences were small as illustrated in Fig. 1, in which the four regression lines almost overlap. This result suggests that *A*_CB_EST_ can be used practically as a surrogate of *A*_CB_ for each PFT.

### Comparison between the pipe- and the qPipe-model allometries using Model II (SMA) regression

**Figure 2:**
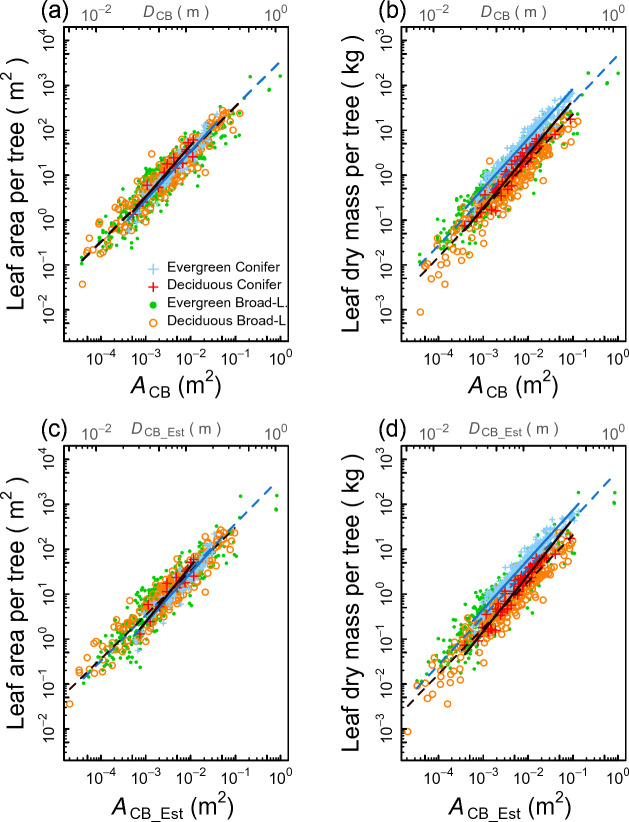
Pipe and qPipe model allometries for the leaf area (LA) and leaf mass (LM) using SMA regression: (**a**) *A*_CB_–LA relationships, (**b**) *A*_CB_–LM relationships, (**c**) *A*_CB_Est_–LA relationships, and (**d**) *A*_CB_Est_–LM relationships. Allometric lines are drawn between horizontal data ranges of respective PFTs, where the blue solid line is for the EC PFT, the black solid line for the DC PFT, the blue dashed line for the EB PFT, and the black dashed line for the DB PFT. The slopes of allometric lines were not significantly different from 1 except for the EC PFT. See Table 1 for coefficients of the power function of each allometry. The significance of the difference in elevations of the regression lines between the four PFTs are given in Table 1.

For the pipe model relationship using SMA regression, the model having ln(*A*_CB_) and PFTs as the predictor variables was selected with the natural-log transformed leaf area (Fig. 2a) and leaf mass (Fig. 2b) as the response variables.

For the leaf area (Fig. 2a), the slopes of allometric lines were not significantly different between the four PFTs (likelihood ratio test, *P* = 0.053), ranging between 1.15 and 1.01 (Table 1). The slopes of broadleaf PFTs (EB, DB) were not significantly different from 1, indicating that the pipe model ratio for the leaf area was constant and the slope follows the pipe model presumption in broadleaf PFTs. In contrast, for conifer PFTs, the difference from 1 was significant in the EC PFT (slope of 1.09, Table 1). The DC PFT had the largest slope (1.15) but the difference from 1 was not significant, probably because only 15 leaf area data were available for the DC PFT, all for Japanese larch (*Larix kaempferi* (Lamb.) Carr*.*) trees. Slopes of the conifer PFTs suggest that their pipe model ratio tended to increase with increasing tree size. Pair-wise comparisons among PFTs showed that the difference between PFTs in the elevation of the allometric line was significant only between DC and EC PFTs (Table 1). Despite these differences, regression lines of the four PFTs almost overlapped (Fig. 2a), indicating that pipe model allometries for leaf area were similar across PFTs.

In contrast, for the leaf mass, the separation of allometric lines was distinct among the four PFTs (Fig. 2b). Differences were significant in both slopes (likelihood ratio test, *P* = 0.018) and intercepts (*P* < 0.000). Elevations of regression lines were greater for conifer (EC, DC) PFTs than for broadleaf (EB, DB) PFTs and were greater for evergreen (EC, EB) PFTs than for deciduous (DC, DB) PFTs [likelihood ratio test for multiple comparison, all *P* < 0.000; Table 1 (2b)]. Furthermore, the slopes tended to be greater for conifer (EC, DC) PFTs than for broadleaf (EB, DB) PFTs (Table 1 (2b)). More importantly, all four PFTs had significant slopes greater than 1, indicating that the pipe model ratio for the leaf mass differs by tree size, with larger trees having a greater pipe model ratio.

The qPipe models that used ln(*A*_CB_EST_) and PFTs as the predictors (Fig. 2c, d) gave results similar to the pipe model (i.e., Fig. 2a, b) with a few exceptions: for leaf area (Fig. 2c), the difference from 1 in the slope was not significant except for the EC PFT (Table 1(2c)). Although differences in elevation were significant between EC and DB PFTs (likelihood ratio test for multiple comparison, *P* < 0.000) and between EC and DC PFTs (*P* = 0.027), the four allometric lines overlapped around the center of the data distribution (Fig. 2c) as in the case of the *A*_CB_–leaf area relationship (Fig. 2a).

For the leaf mass, the qPipe model had clear differences in regression lines (Fig. 2d), as in the case of the pipe model (Fig. 2b). Elevations were significantly different among PFTs (likelihood ratio test, *P* < 0.000) with conifer PFTs (EC, DC) having higher elevations than broadleaf (EB, DB) PFTs (likelihood ratio test for multiple comparison, *P* < 0.000) as in the pipe model (Table 1(2d)). Additionally, the slopes tended to be greater for conifer (EC, DC) PFTs than for broadleaf (EB, DB) PFTs and were significantly different from 1 except for the DB PFT (Table 1(2d)).

In summary, for the leaf area, both the pipe model and qPipe gave regression slopes closer to 1, with little apparent difference in the allometric line among PFTs. For the leaf mass, in contrast, slopes of the four PFTs were greater than 1 in both the pipe model and the qPipe model, and their allometric lines tended to differ in elevation.

### Prediction of the tree leaf area using the qPipe model allometry

**Figure 3:**
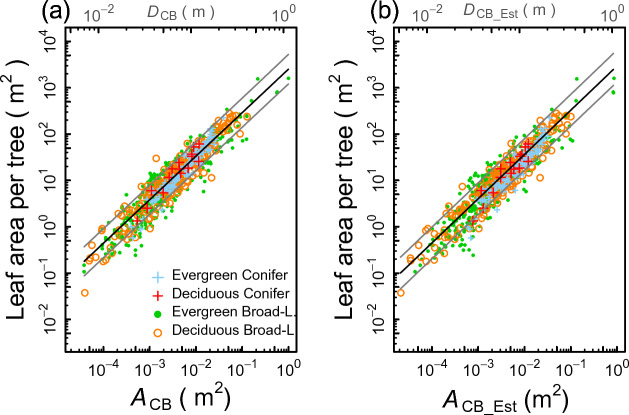
Pipe and qPipe model allometries for the leaf area (LA) obtained using GLMMs: (**a**) *A*_CB_–LA relationship of the pipe model and (**b**) *A*_CB_Est_–LA relationship of the qPipe model. In both cases, models including the PFT as a fixed effect were not selected, such that a single allometric line and its 95% prediction intervals for log-transformed values of LA are drawn by pooling all data of the PFTs. Species are designated as the random effect for the elevation of the regression line. See Table 1 for coefficients of each allometric equation, Table 2 for the coefficients of the equation approximating the 95% prediction intervals, and Supplementary Information S1 and S2 for the method of approximating the prediction intervals equations.

To predict the tree leaf area, the allometric relationship of the qPipe model was obtained through model selection with GLMMs (Fig. 3b). For comparison, the results of the pipe model using GLMMs (Fig. 3a) are also presented. In both cases, the PFT was not selected as a predictor of the Model I regression, in contrast with the results of Model II regression (Fig. 2). Selected models were those with ln(*A*_CB_) or ln(*A*_CB_EST_) as the fixed effect variable and species as the random intercept term (Fig. 3a, b, Table 1). This indicates that, for predicting leaf area using *A*_CB_ or *A*_CB_EST_, differences in the PFT are negligible as far as our collective data sets are concerned. In other words, the qPipe model (Fig. 3b) is applicable to the prediction of the leaf area of a tree without the need to consider differences in the PFT. Note, however, that different species would have different elevations of the allometric line as the species was selected as the random effect on the elevation. Ninety-five percent prediction intervals are drawn in Fig. 3 using an approximating equation, which is given in Table 2 (see also Supplementary Information S2). The intervals are seen to be slightly wider for the qPipe model (Fig. 3b) than for the pipe model (Fig. 3a).

**Table 2:** Coefficients of the equation for 95% prediction intervals of the predicted ln(LA) or ln(LM) for Figs. 3 and 4.

Figure number	Response variable	Predictor variable	PFT(s)	C_0_	C_1_	C_2_	C_3_	C_4_	Residual SE
3a	ΔPI_95%_ for ln(LA)	ln(*A*_CB_)	Total PFTs	0.71655	0.00590	0.00052	–	–	0.00002
3b		ln(*A*_CB_Est_)	Total PFTs	0.77894	0.00622	0.00054	–	–	0.00002
4a	ΔPI_95%_ for ln(LM)	ln(*A*_CB_Est_)	EC	0.48920	0.01772	0.00181	–	–	0.00002
4b			DC	2.004	0.3389	57	− 5.206	62.89	–
4c			EB	0.78223	0.01286	0.00112	–	–	0.00007
4d			DB	0.81205	0.01924	0.00159	–	–	0.00007

**Figure 4:**
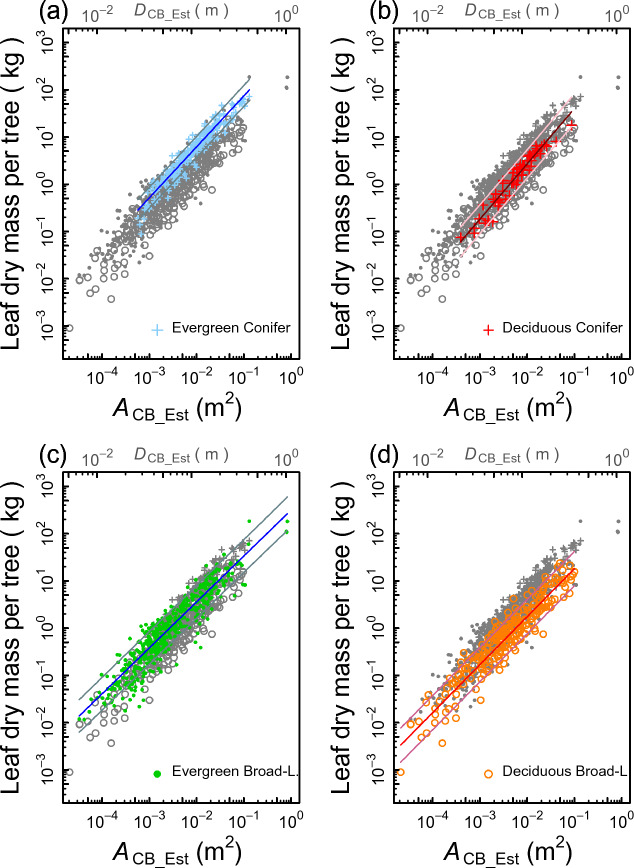
qPipe model allometries between *A*_CB_Est_ and the leaf mass (LM) for (**a**) EC, (**b**) DC, (**c**) EB and (**d**) DB PFTs. The allometry obtained using the GLMM was selected in (**a**), (**c**), and (**d**), where species were designated as the random effect for the elevation of the regression line. For (**b**), an ordinary linear regression model pooling all species data was selected. See Table 1 for coefficients of each allometric equation, Table 2 for the coefficients of the equation approximating the 95% prediction intervals, and Supplementary Information S1 and S2 for the method of determining/approximating the prediction interval equations.

Except for Fig. 4b, the upper and lower 95% prediction intervals of an estimate of LA* or LM* (= *Y* ) for a given ln(*A*_CB_*) or ln(*A*_CB_Est_*) (= *X*) are given by exp[ln(*Y*) ± ΔPI_95%_], where ΔPI_95%_ = (C_0_ + C_1_ × *X* + C_2_ × *X*^2^). For Fig. 4b, ΔPI_95%_ for ln(*Y*) is calculated with the formula of the 95% prediction interval in ordinary least-squares regression; i.e., ΔPI_95%_ = {C_0_ × C_1_ × sqrt[1 + 1/C_2_ + (ln(*X*) − C_3_)^2^/C_4_]}.

### Prediction of the tree leaf mass using the qPipe models

The results presented in Fig. 2b, d clearly show that, in predicting the tree leaf mass, the four PFTs have different qPipe model allometry equations. Hence, the regression calculation was performed for each PFT independently, allowing the calculation of prediction intervals. Models with species as the random intercept term were selected for the EC, EB, and DB PFTs (Fig. 4a, c, d) whereas ordinary least-squares (LS) linear regression without a random effect was selected for the DC PFT (Fig. 4b, Table 1). Consistent with the results of Model II regression (Fig. 2d), the elevation of the allometric line tended to be greater for the evergreen trees than for the deciduous trees in both conifer and broadleaf PFTs (Table 1).

### Example of the comparison of the pipe and qPipe models for the LAI

**Figure 5:**
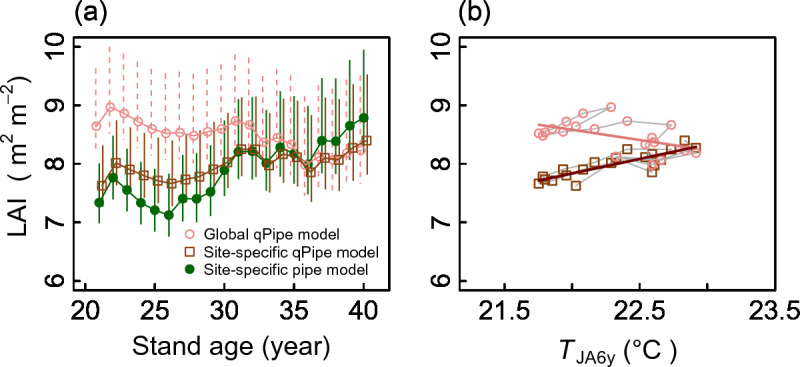
Comparison of three allometric methods in estimating the leaf area index (LAI) for a hinoki cypress (*Chamaecyparis obtusa*) stand. (**a**) 20-year changes in the LAI of the stand obtained using the three methods. Each of the three allometries takes the form LA = CF × Elevation × *X*^Slope^, where LA (m^2^) is the leaf area per tree, *X* represents *A*_CB_ (the site-specific pipe model) or *A*_CB_Est_ (the site-specific and global qPipe models) of a tree, and CF is the factor correcting for bias regarding log-transformed regression. The values of the CF, elevation and slope differ between the three methods. See Supplementary Fig. S1 and Supplementary Table S1 in Supplementary Information S1 for coefficients of these allometries. For the site-specific pipe and qPipe models, allometric equations were formulated using 47 trees sampled near the stand where the LAI was estimated^8^. The allometry of the global qPipe model is the same as in Fig. 3b and Table 1(3b). LA for each tree each year in the hinoki stand was calculated by substituting measurements of *A*_CB_ or *A*_CB_Est_ of each tree each year into the three different allometries. The LAI for each year was then calculated by taking their sum and dividing by the stand area. (**b**) Relationship between the LAI of each year and the moving average of the mean temperature in July and August for the last 6 years (*T*_JA6y_), where the LAI was calculated using the site-specific qPipe allometry (open squres) or the global qPipe allometry of Fig. 3b (open circles). In the original paper, a significantly positive relationship was obtained^8^. For coefficients of the regression, see Supplementary Table S1 in Supplementary Information S1.

Lastly, the three methods of stand LAI estimation were compared. A 20-year change in the LAI of an evergreen conifer hinoki (*C. obtusa*) stand was estimated using the measured *A*_CB_ of each tree and the original^8^ site-specific pipe model allometry prepared for the particular stand, the calculated *A*_CB_Est_ and the site-specific qPipe model allometry, and the calculated *A*_CB_Est_ of each tree and the global qPipe model allometry for the leaf area (shown in Fig. 3b). The LAI estimated with the site-specific qPipe model showed interannual changes similar to those of the LAI estimated using the site-specific pipe model, though there were differences (but not significant) in the LAI (Fig. 5a). A positive relationship between the LAI and 6-year running average of the mean summer temperature, which has been reported previously^8^, was reproduced with the site-specific qPipe allometry (Fig. 5b). However, the LAI estimated with the global qPipe allometry could not reproduce the relationship (Fig. 5a, b), especially at earlier stand ages. These results demonstrate that qPipe allometry is useful if the site-specific qPipe allometry is available together with measurements of the tree height, crown-base height, and DBH for each tree, whereas the global qPipe allometry may need to be carefully considered when applied to the LAI estimation of an unknown stand, as we discuss later.

## Discussion

### Leaf area versus leaf mass

Although the main objective of the present study is to examine the applicability of the qPipe model, the study also advances our understanding of the pipe model. An important presumption of the original pipe model is that the amount of leaves per unit cross-sectional area of the trunk at the crown base, or the pipe model ratio, is constant^13^. We show that this appears to hold for the leaf area but not for the leaf mass, as long as the stem cross-sectional area *A*_CB_ is considered as a predictor variable. In the Model II regression analysis (Fig. 2a, Table 1), the slope of approximately 1 for PFTs except for the EC PFT indicates that the pipe model ratio for the leaf area (leaf area per unit *A*_CB_) was statistically constant irrespective of *A*_CB_. In contrast, for the leaf mass, the slope of the Model II regression was significantly greater than 1 for each PFT (Fig. 2b, Table 1). This indicates that the pipe model ratio for the leaf mass (leaf mass per unit *A*_CB_) increased with increasing *A*_CB_. The difference in the slope of a PFT between the leaf area and leaf mass can be ascribed to a tendency that taller trees tend to have a greater tree LMA than shorter trees, as mentioned in the Introduction.

Figure 2a also shows an interesting result for the leaf area that there was little difference in the pipe model allometries, if at all, between PFTs. This implies that the pipe model ratio for the leaf area remained approximately constant irrespective of the PFT as long as *A*_CB_ is used as the denominator of the pipe model ratio. However, the use of the sapwood area in the stem cross section, rather than the stem cross-sectional area *A*_CB_, has been regarded as a reason why the pipe model holds, as the former can biologically explain the hydraulic functioning^15,16^. Additionally, the xylem structure for the water transport system is known to vary between the four PFTs^27,28^. For example, the gymnosperm (EC and DC) species depend on tracheids for water transport and do not have vessels, unlike angiosperm (EA and DA) species. Even between angiosperm species, the vessel structure of the xylem on the stem cross-section varies; e.g., between ring-porous wood (e.g., some deciduous oak spp.), diffuse-porous wood (some beech spp.), and radial-porous wood (some evergreen oak spp.). Considering these possible variations in xylem hydraulic properties, it seems curious that the slope of approximately 1 held for the leaf area–*A*_CB_ relationship with almost no difference among the four PFTs. We do not have an explanation for this as yet. Considering that the leaf area rather than the leaf mass is likely the primary quantity of leaves relating to photosynthesis^20,29^, trees may adjust to maximize water conduction efficiency for photosynthesis and transpiration irrespective of the xylem structure^30^, which may have resulted in a convergence to a similar proportionality among PFTs in the leaf area–*A*_CB_ relationship.

For the pipe model relationship to hold, stem thickening growth must occur in such a way that the stem cross-sectional area or sapwood area is coordinated to be proportional to the leaf amount. However, it is not known what types of signal are involved in this coordination. One possibility would be the effect of the plant hormone auxin (indole-3-acetic acid, IAA), which plays an important role in determining the radial growth of a stem by affecting the activity of cell division in the cambial meristem^31^. As IAA is produced in apical buds and growing shoots in the crown and transported to the cambial region of the stem, the IAA level in the cambial region is known to be affected by the crown size^32–35^. This may be a cue to the connection between the leaf amount and the stem cross-sectional area. Meanwhile, a twig-scale study suggested a mechanism that allows the pipe model relationship to hold in terms of the source–sink relationship^36^. Further study combining several biological mechanisms may be necessary to solve the abovementioned questions.

### How and when does the qPipe model work?

**Figure 6:**
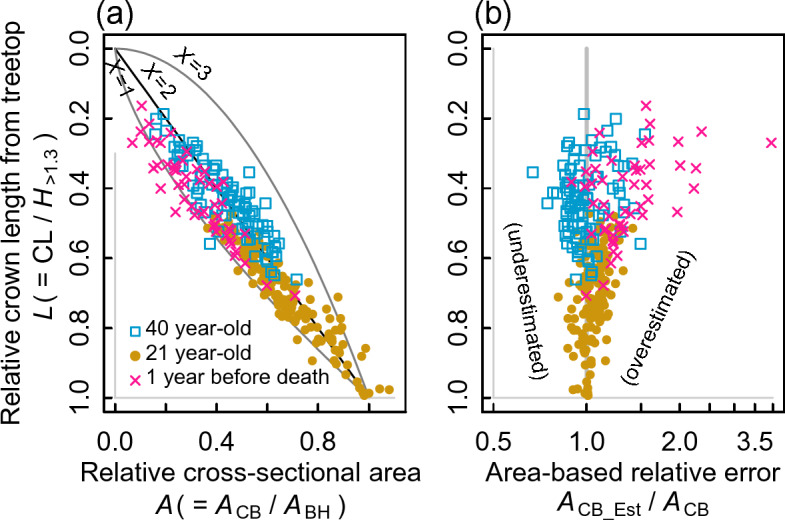
Measurements of *A*_CB_ and the crown length compared with the stem taper curve assumed with the qPipe model. Tree data from published studies^8,9^ were used in the analysis. (**a**) Relative crown length and *A*_CB_ relative to *A*_BH_ for each live tree at 21 (filled circles) and 40 (open squares) years of stand age in an even-aged population of a hinoki cypress stand^8,9^. *X* = 1, 2 and 3 corresponds to the stem shape of a cone, paraboloid of revolution (Eq. (2)), and cubic solid of revolution, respectively. As the line of *X* = 2 corresponds to the qPipe model, data points on the line of *X* = 2 imply that *A*_CB_ and *A*_CB_Est_ estimated by the qPipe model are in agreement. Pink crosses represent data of trees that died between 21 and 40 years of age; the marks are the data 1 year before their death. Such trees are found to be overestimated. (**b**) Relative error of the stem cross-sectional area at the crown base (= *A*_CB_Est_*/A*_CB_).

Figures 1 and 2 show that the qPipe model provided allometry comparable to that obtained using the pipe model. However, whether the qPipe model can fairly estimate the actual *A*_CB_ or not depends on the actual form of the tree stem. Here, we explain this using the stem taper equation, a mathematical expression of the change in stem thickness from breast height to treetop. In the field of forest sciences, the shape of a tree stem is often expressed by a simple equation^37^. The relative taper of the stem between the crown base and breast height is expressed as

3 $$A = L^{{{2}/X}} ,$$

where *L* is the distance from the treetop along the stem (relative scale; 0 < *L* ≤ 1, where *L* = 1 at breast height), *A* is the stem cross-sectional area at *L* (relative scale; 0 < *A* ≤ 1, where *A* = 1 at breast height), and *X* is a scaling parameter indicating the degree of stem taper. The stem shapes of a cone, paraboloid of revolution, and cubic solid of revolution correspond to *X* = 1, 2, and 3, respectively (curved lines in Fig. 6a). As mentioned in the Introduction, the calculation of *A*_CB_Est_ in the qPipe model is based on the assumption of a paraboloid of revolution; i.e., *X* = 2 in Eq. (3). It follows that, if the qPipe model is applied to a tree stem having *X* < 2, then *A*_CB_Est_ > *A*_CB_ (i.e., an overestimation, as *L*^2/2^ > *L*^2/*X*^), and if applied to trees having *X* > 2, then *A*_CB_Est_ < *A*_CB_ (i.e., an underestimation, as *L*^2/2^ < *L*^2/*X*^) (Fig. 6a). Hence, concordance between *A*_CB_ and *A*_CB_Est_ (Fig. 1) depends on the stem shape that a tree actually had, or the *X* value in Eq. (3). This is a possible source of estimation error of *A*_CB_Est_ for an individual tree. However, in estimating the total leaf amount of a stand, what should be considered further is the balance between overestimation and underestimation among the trees in the stand. The stand-level leaf amount may be overestimated if there are many trees with *X* < 2 and underestimated if there are many trees with *X* > 2.

The data points of Fig. 6a illustrate an example of actually measured *A*_CB_ for all trees in an even-aged evergreen conifer stand at 21 and 40 years of stand age^8,9^. At 21 years of stand age, a majority of the trees had *A* (≡ *A*_CB_/*A*_BH_) at the crown base in the region *X* < 2 (Fig. 6a), indicating that their *A*_CB_Est_ was an overestimation of the actual *A*_CB_ (Fig. 6b). This tendency changed at 40 years of stand age, when there were still some overestimated trees but many underestimated trees also (Fig. 6a, b). This change is ascribed to a change in the stem taper with tree growth under competition. The good estimation of the LAI by the site-specific qPipe model with non-significant differences in the LAI (Fig. 5a) was partly due to the overestimation and underestimation offsetting each other between trees. It is likely that trees of a stand vary in terms of *X* in Eq. (3).

A study of four conifer species in North America^37^ reported that there were more trees with *X* = 2 than those with *X* = 3. This may suggest that the assumption of *X* = 2 in the qPipe model is likely a moderate assumption for estimating the stand LAI due to the presence of trees with both *X* < 2 and *X* > 2 in a stand. Additionally, the qPipe model provides over/underestimations due to other factors. For example, many long-lived ‘giant’ trees^38,39^ are known to undergo treetop breakage because of natural phenomena such as lightning strikes and strong winds. Such trees are expected to have a less tapered, cylindrical stem (i.e., *X* > 2 in Eq. (3)) owing to the loss of their top part and would have a much higher *H* value if breakage had not occurred. The leaf amount for such top-broken trees may result in underestimation with the qPipe model. Another situation that may lead to error is a species having leaf turnover without turning over branches; evergreen conifers such as black spruce (*Picea mariana* (Mill.) B.S.P.) turnover needled shoots with epicormic branching (re-sprouting of new shoots) on a primary branch whereas older shoots on the same branch wither and fall^40,41^. For such a tree, the amount of leaves per tree may be in a steady state, whereas *A*_CB_ may increase annually without a change in its crown-base location. If this happens, the leaf amount per unit *A*_CB_ should decrease with age. These problems apply to not only the qPipe model but also the pipe model. The applicability of the qPipe model and pipe model needs to be carefully considered for trees having an atypical crown structure.

## Conclusion

With our collective dataset, the constant pipe model ratio assumed in the pipe model held for LA but not for LM, which likely reflects changes in the tree LMA with tree size. We proposed qPipe model allometry considering the difference in PFTs, where measurements of the tree height, crown-base height, and DBH are used in estimating the leaf area and leaf mass of an individual tree. As these measurements can be made in the field and the 95% confidence intervals of the estimated individual leaf area or mass are calculable, the stand LAI and its confidence intervals can be estimated for a stand for which the stand-specific allometry of the leaf area or leaf mass is not available, as is often the case in large-scale studies. Although data for ascertaining the applicability of the qPipe model to LAI estimation are still limited, we believe that the investigation of the applicability of the present method is worth continuing in future work.

## Materials and methods

### Data sources

Many of the data of the present study are taken from the BAAD dataset^42^, which is a meta-dataset comprising many published/unpublished data of individual trees. However, while choosing data suitable for the present study, we found that a part of the BAAD data had been incorrectly copied from original data. We referred to the original data source in adding/correcting such data. Additionally, other data sources were collected and combined with the BAAD data.

With the combined data, we selected individual trees that satisfied the conditions of a tree height greater than 1.3 m, breast height of at least 1.3 m, crown-base height of at least breast height, either the stem diameter or cross-sectional area being recorded for both the breast height and crown-base height, and either the leaf area (LA, m^2^) or leaf dry mass (LM, kg) being recorded for an individual tree. In the process of regression calculation, we carried out Grubb’s test (R package “outliers”^43^) to remove outliers from each of the ln(*A*_CB_)–ln(LA) and ln(*A*_CB_)–ln(LM) relationships until no outliers remained. If necessary, Grubbs’ test was conducted for each PFT or each species. Data for palm trees (monocots) were excluded. The list of all of the data used in the analyses of the present study is given as the Supplementary file “AllData.csv”, the explanation of which is given in Supplementary Information S4.

### Data analyses

All statistical calculations were made using R^26^. For Model II regression (Figs. 1, 2), the SMA regression function available from the package smatr^44^ in R was used, where the “robust” setting was adopted to lessen the effects of outliers. Model selection was performed using the AIC. As the current version of smatr does not allow the calculation of random effects, PFTs were treated as the predictor by pooling all species in each PFT. In Model I regression used for predictions (Figs. 3, 4), ordinary least-squares or the mixed models package lme4^45^ was used, both of which allow the calculation of prediction intervals. In GLMMs with log-transformed variables, a Gaussian error distribution was chosen for normality. Models with the PFT as either a fixed effect or random effect were compared to select the best models using the AIC. The species in each PFT were treated as a random effect, where model selection was made by comparing models including species only in a random-effect intercept term with those including species in both intercept and slope terms. Some models with error messages (e.g., the model failed to converge) were discarded irrespective of the AIC. As a result, in all mixed models of the present study (Figs. 4, 5a, c, d), the species were treated as the only intercept term of the random effect.

Note that the prediction intervals for the mixed models in Figs. 3 and 4 were first calculated with lme4. The prediction intervals were then approximated with the quadratic equation function of* A*_CB_ or *A*_CB_Est_ (Table 2) to allow the calculation of prediction intervals for other studies. This approximation was performed using the non-linear regression method nls() in R. An example of the results of calculation is given in Supplementary Fig. S2.

As the regression calculation for Model I regression (Figs. 3, 4) was made through natural-log transforming the predictor and the responses of a power function, the bias arising from the log-transformation was corrected by multiplying the power function with a correction factor CF^12,46^. The CF values are given in Table 1. See also equation (S5) in Supplementary Information S1 for an example of the equation.

## Supplementary Information

Supplementary Information 1.

Supplementary Information 2.

## Data Availability

The data used in the present study is available as Supplementary Information File S1.
