# Supplementary material for: Allometry of the quasi-pipe (qPipe) model for estimating tree leaf area and tree leaf mass applied to plant functional types
Source: Sci Rep. 2023 Jun 19;13:9954. doi: 10.1038/s41598-023-37112-1 (PMC10279641; doi:10.1038/s41598-023-37112-1)
Supplement: Supplementary file 1 — Supplementary Information 1. [file 41598_2023_37112_MOESM1_ESM.docx]

**Supplementary Information for:**

**Allometry of the quasi-pipe (qPipe) model for estimating tree leaf area and tree leaf mass applied to plant functional types**

Akihiro Sumida ^1,*^, Yoshiyuki Inagaki ^2, *^, Takuya Kajimoto^3^, Masumi Katsuno-Miyaura^4^, Akira Komiyama^5^, Nahoko Kurachi^4^, Tomiyasu Miyaura^6^, Shigeaki F. Hasegawa^7^, Toshihiko Hara^8^, Kiyomi Ono^8^, Masahito Yamada^8^

1 Graduate School of Life & Environmental Sciences, Kyoto Prefectural University, 1-5 Shimogamohangi, Sakyo-ku, Kyoto 606-8522, Japan;

2 Shikoku Research Center, Forestry and Forest Products Research Institute, 2-915 Asakuranishi, Kochi 780-8077, Japan;

3 Sado Island Center for Ecological Sustainability, Niigata University, 94-2 Koda, Sado, Niigata 952-2206, Japan;

4 Hiraoka Forest Research Institute, Aoyama, Otsu, Shiga 520-2101, Japan

5 Gifu University, 1-1 Yanagido, Gifu 501-1193, Japan;

6 Faculty of Advanced Science and Technology, Ryukoku University, Seta Oe-cho, Otsu, Shiga 520-2194, Japan;

7 Faculty of Human Sciences and Cultural Studies, Yamanashi Eiwa College, 888 Yokonemachi, Kofu, Yamanashi 400-8555, Japan;

8 Institute of Low Temperature Science, Hokkaido University, N19W8, Sapporo 060-0819, Japan

* Corresponding author: asumida@kpu.ac.jp, yinagaki@affrc.go.jp

**S1. Allometries and regressions concerning Fig. 5 in the main text**

The site-specific pipe model for the leaf area of *Chamaecyparis obtusa* is given by

LA = CF exp(9.37) *A*_CB_^1.23^, (S1)

where LA is tree leaf area (m^2^), *A*_CB_ (m^2^) is the stem cross-sectional area at the crown base obtained using a dataset (see refs^S1^), and CF (= 1.06) is a factor for correcting the bias concerning the log-transformation of LA and *A*_CB_. CF is given by

CF = exp(*s*^2^/2), (S2)

where *s* is the standard error of regression. See Supplementary Table S1(5b) for other coefficients of this regression.

The upper and lower limits of the 95% prediction intervals (PIs) of LA are calculated ^6^ as

$$\mathrm{PI}\left( \mathrm{LA}_{\left（ {A_{\mathrm{CB}}}^{*} \right）} \right)=\left\{ \ln\left( \mathrm{LA}_{\left（ {A_{\mathrm{CB}}}^{*} \right）} \right)\pm t_{\left( \frac{\alpha}{2},n-2 \right)}\times X \right\}, （S3）$$

where ${A_{L}}_{\left（ {A_{\mathrm{CB}}}^{*} \right）}$ is the predicted value of LA when $A_{\mathrm{CB}}$= ${A_{\mathrm{CB}}}^{*}$, *n* (= 47) is the sample size, and $t_{(\alpha/2, n-2)}$ (= 2.01) is the *t* value at a significance level *α* (= 0.05). *X* is given by ^6^

$X=s\times\sqrt{1+\frac{1}{n}+\frac{{\left[ {\ln(A_{\mathrm{CB}}}^{*} \right)-\bar{\ln\left( A_{\mathrm{CB}} \right)} ]}^{2}}{\sum{{[ ln \left( A_{\mathrm{CB}} \right)}_{i}-\bar{\ln\left( A_{\mathrm{CB}} \right)} ]}^{2}}}$ , (S4)

where *s* (= 0.318) is the residual standard error of regression and $\bar{\ln(A_{CB})}$ is the mean value of $\ln(A_{CB})$ (= −5.76).

The site-specific qPipe model for the leaf area of *C*. *obsusa* is given by

LA = CFq exp(9.51) *A*_CB_EST_^1.28^, (S5)

where LA is the tree leaf area (m^2^), *A*_CB_Est_ (m^2^) is the predictor calculated with equation (2) in the main text, and CFq (= 1.06) is the factor for correcting the bias concerning log-transformation of LA and *A*_CB_Est_. The upper and lower limits of the 95% prediction intervals (PIs) of LA were calculated^6^ as in equations (S4) and (S5), but *s* = 0.333 and $\bar{\ln(A_{CB\_EST})}$ = −5.67.

The allometric lines for the pipe (S1) and the qPipe models (S3) are illustrated in Supplementary Fig. S1.

**Supplementary Reference S1**.

S1 Hagihara, A., Yokota, T. & Ogawa, K. Allometric relations in hinoki (*Chamaecyparis obtusa* (Sieb. et Zucc.) Endl.) trees. *Bull. Nagoya Univ. Forest* **12**, 11–29, http://hdl.handle.net/2237/8694 (1993).


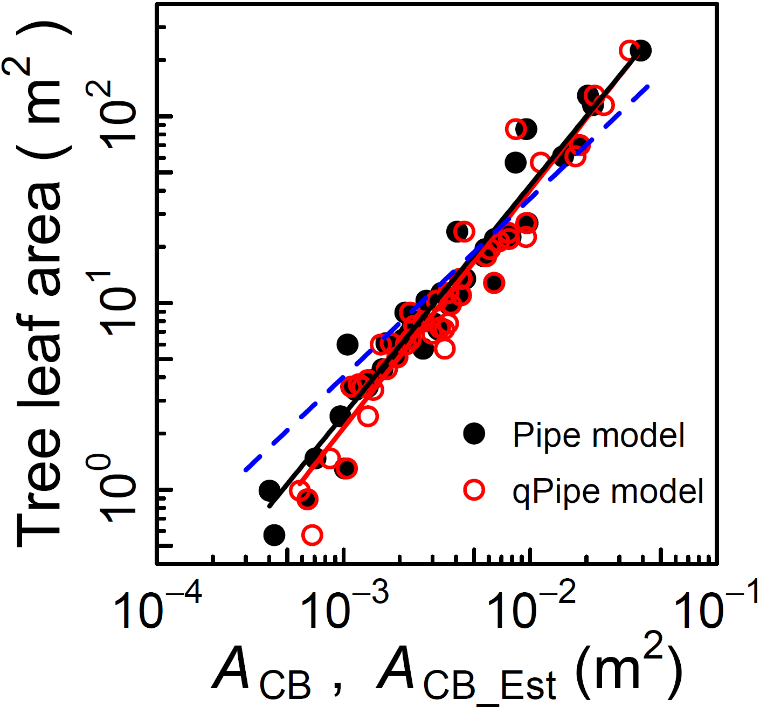


**Fig. S1.** Pipe and qPipe model allometries for 47 sampled trees of *Chamaecyparis obtusa*. The black solid line corresponds to the pipe model allometry and the red solid line to the qPipe model allometry. These allometry data^S1^ are included in Supplementary File S1, “AllData.csv”. See Supplementary Table S1 for regression coefficients. The global qPipe model allometry, which is the same as that in Fig. 3b, is drawn with the blue dashed line for comparison. Note that two different explanatory variables, *A*_CB_ (filled circles) and *A*_CB_Est_ (open circles), of each tree are shown, and the two explanatory variables are not exactly the same. In a preceding study^12^, the tree leaf area for trees whose *A*_CB_ could not be directly measured by climbing the tree was calculated through the pipe model allometry by substituting *A*_CB_Est_ into the pipe model allometric equation. However, in the present study, the tree leaf amount is calculated by substituting *A*_CB_ or *A*_CB_Est_ into the pipe or qPipe model allometry, respectively. The LAIs of a stand estimated using the three allometric relationships in Fig. S1 are presented in Fig. 6.

| Figure number | Type of allometry | Target species of allometry | Response variable | Predictor　variable | Type of regression | Elevation | Slope | CF | *R* 2 | *F* | *P* | *n* |
| --- | --- | --- | --- | --- | --- | --- | --- | --- | --- | --- | --- | --- |
| 5a | Global qPipe  model | All PFTs | ln(LA) | ln(*A* CB_Est) | GLMM | 2742 | 0.955 | 1.077 | – | – | – | 768 |
|  | Site-specific  pipe model | *C. obtusa* | ln(LA) | ln(*A* CB_Est) | least square | 13550 | 1.275 | 1.057 | 0.937 | 671.3 | <0.000 | 47 |
|  | Site-specific  qPipe model | *C. obtusa* | ln(LA) | ln(*A* _CB_) | least square | 11700 | 1.230 | 1.052 | 0.943 | 743 | <0.000 | 47 |
| 5b | Site-specific  qPipe model | – | LAIS | *T* JA6y | least square | −5.72 | 0.583 | – | 0.769 | 59.83 | <0.000 | 20 |
|  | Global qPipe  model | – | LAIG | *T* JA6y | least square | 16.3 | −0.349 | – | 0.248 | 5.93 | 0.025 | 20 |

**Table S1.** Coefficients of allometries and regression concerning Fig. 5. For Fig. 5a, coefficients of the equation LA = CF × Elevation × *X*^Slope^ are given, where *X* represents *A*_CB_ or *A*_CB_Est_. The three allometric lines are illustrated in Fig. S1. The coefficients of the global qPipe model are the same as those in Fig. 3b. For Fig. 5b, the results of regression *Y* = Elevation + Slope × *X* are shown, where *Y* represents LAI_S_ (the LAI calculated with the site-specific qPipe allometry) or LAI_G_ (the LAI calculated with the global qPipe allometry), and *X* represents *T*_JA6y_. See the main text for abbreviations and units.

**S2. Equation for 95% prediction intervals of allometry using general linear mixed models**

The 95% prediction intervals for a regression equation of the GLMM were calculated using the ‘predict’ method in the library lme4 ver. 1.1-30 in R. The prediction intervals at the population level were determined by setting all random effects values to zero ^39^. However, the obtained prediction intervals are difficult to apply to other studies as the intervals are directly calculated on our personal computer without using a specific function of *A*_CB_ or *A*_CB_Est_. Hence, we explored an equation approximating each prediction interval line as follows.

In the ordinary least-squares method, prediction intervals of a given predictor variable of a regression are calculated using equation (S4), which is given by the square root of a quadratic function of the predictor, ln(*A*_CB_). Practically, we found that the prediction intervals calculated with lme4 could be approximated with a quadratic function. Here, we take this as an example of the relationship between the leaf area (LA) and *A*_CB_ for the GLMM (Fig. 3a). The actual data range of ln(*A*_CB_) was divided into the same intervals to obtain 20 hypothetical predictor values (ln(*A*_CB_)*_i_*; *i* = 1 to 20), and its natural-log-transformed upper or lower half of the 95% prediction interval (${\Delta PI}_{i}$) from the mean estimate of ln(${\hat{\mathrm{LA}}}_{i}$)) at each ln(*A*_CB_)*_i_* was calculated with the predict methods of lme4. For the 20 (ln(*A*_CB_)*_i_*, ΔPI*_i_*) dataset, the relationship was then approximated according to

${\Delta PI}_{i}$= C_0_ + C_1_ ln(*A*_CB_)*_i_* + C_2_ ln(*A*_CB_)*_i_* ^2^ , (S6)

where C_0_, C_1_, and C_2_ are coefficients (Table 2 in the main text) estimated with a non-linear regression method of R.

Note that the antilog values of the upper and lower 95% prediction intervals (PI*_i_*) for a given value of ${\hat{\mathrm{LA}}}_{i}$ are then given as

$\mathrm{PI}_{i}=\exp\left( \ln\left( {\hat{\mathrm{LA}}}_{i} \right)\pm{\Delta PI}_{i} \right). （S7）$

As an example, the upper ΔPI obtained with the ‘predict’ method (Predicted ΔPI) for Fig. 3a and its approximated ΔPI obtained using equation (S6) are shown in Supplementary Fig. S2.


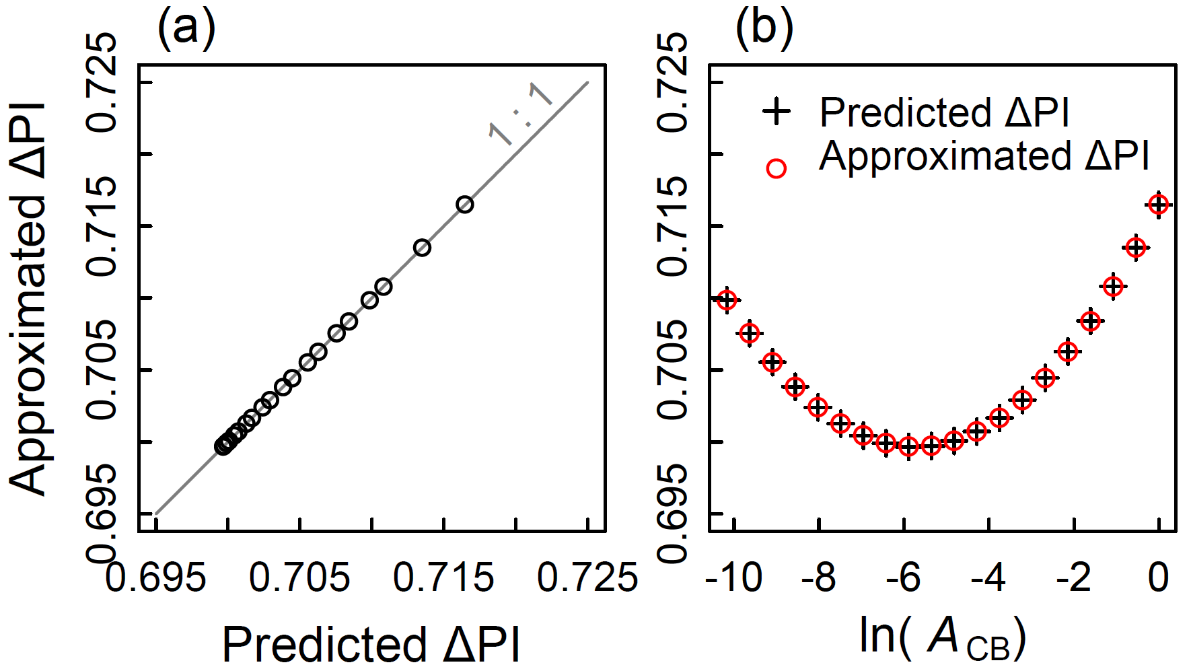


**Fig. S2.** Comparison of the upper half of the prediction intervals obtained using the prediction method of lme4 (Predicted ΔPI) and that approximated with equation (S6) (Approximated ΔPI). (**a**) Relationship between the Predicted ΔPI and the Approximated ΔPI expressed as the upper half of the natural-log transformed 95% prediction intervals from each leaf area estimate, ln(${\hat{\mathrm{LA}}}_{i}$). The 1:1 diagonal line is drawn. (**b**) Predicted ΔPI and Approximated ΔPI plotted against the 20 ln(*A*_CB_)*_i_* values.

**S3. List of species and number of trees of each species**

Note that there are species whose species names were unknown in the dataset from tropical rainforest. In that case, domestic common names are listed. Note also that the list includes species that were unknown/unidentified (Species IDs 154 and 155).

| Species ID | Species name | Number  of trees |
| --- | --- | --- |
| 1 | *Abies firma* | 1 |
| 2 | *Abies sachalinensis* | 35 |
| 3 | *Abies veitchii* | 11 |
| 4 | *Acer palmatum* | 2 |
| 5 | *Acer pictum* | 11 |
| 6 | *Acer sieboldianum* | 8 |
| 7 | *Ang kumo* (Schima) | 1 |
| 8 | *Angkot khmau* (Diospyros) | 1 |
| 9 | *Anquin suwa* (Saraca) | 11 |
| 10 | *Aporosa elmeri* | 6 |
| 11 | *Aporosa sphaedophora* | 2 |
| 12 | *Artocarpus anisophyllus* | 1 |
| 13 | *Baccaurea deflexa* | 1 |
| 14 | *Baccaurea kunstleri* | 1 |
| 15 | *Baccaurea pendula* | 2 |
| 16 | *Baccaurea* sp. | 3 |
| 17 | *Barringtonia macrostachy* | 1 |
| 18 | *Beilschmiedia* sp. | 1 |
| 19 | *Betula ermanii* | 29 |
| 20 | *Betula grossa* | 4 |
| 21 | *Betula maximowicziana* | 7 |
| 22 | *Betula platyphylla* | 5 |
| 23 | *Camellia japonica* | 40 |
| 24 | *Camellia lutchuensis* | 1 |
| 25 | *Carpinus cordata* | 4 |
| 26 | *Carpinus japonica* | 3 |
| 27 | *Castanea crenata* | 6 |
| 28 | *Castanopsis cuspidata* | 34 |
| 29 | *Castanopsis sieboldii* | 3 |
| 30 | *Cham cha chumoal (Pedocarpus)* | 1 |
| 31 | *Chamaecyparis obtusa* | 98 |
| 32 | *Chang ko koh (Myristica)* | 1 |
| 33 | *Chengiopanax sciadophylloides* | 1 |
| 34 | *Chu dai* | 2 |
| 35 | *Chuwai huowat* | 2 |
| 36 | *Cinnamomum tenuifolium* | 1 |
| 37 | *Clethra barbinervis* | 21 |
| 38 | *Cleyera japonica* | 2 |
| 39 | *Cornus controversa* | 8 |
| 40 | *Cryptomeria japonica* | 32 |
| 41 | *Dacryodes rugosa* | 1 |
| 42 | *Daphniphyllum macropodum* | 1 |
| 43 | *Diabelia serrata* | 2 |
| 44 | *Dialium indum* | 3 |
| 45 | *Dialium platycephalum* | 1 |
| 46 | *Dialium* sp. | 1 |

| ID | Species name | Number  of trees |
| --- | --- | --- |
| 47 | *Dillenia excelsa* | 1 |
| 48 | *Diospyros kaki* | 1 |
| 49 | *Dipterocarpus crinitus* | 1 |
| 50 | *Distylium racemosum* | 4 |
| 51 | *Dryobalanops* sp. | 1 |
| 52 | *Drypetes* sp. | 2 |
| 53 | *Elaeocarpus* sp. | 1 |
| 54 | *Eucalyptus nitens* | 35 |
| 55 | *Eugenia cuprea* | 2 |
| 56 | *Eugenia* sp. | 1 |
| 57 | *Eurya japonica* | 48 |
| 58 | *Fagus crenata* | 11 |
| 59 | *Fagus japonica* | 5 |
| 60 | *Frangula crenata* | 2 |
| 61 | *Fraxinus mandshurica* | 1 |
| 62 | *Fraxinus sieboldiana* | 1 |
| 63 | *Girroniera nervosa* | 1 |
| 64 | *Hamamelis japonica* | 3 |
| 65 | *Hopea mangerawan* | 4 |
| 66 | *Horsfieldia grandis* | 1 |
| 67 | *Ilex chinensis* | 2 |
| 68 | *Ilex crenata* | 2 |
| 69 | *Ilex integra* | 1 |
| 70 | *Ilex macropoda* | 4 |
| 71 | *Ilex pedunculosa* | 16 |
| 72 | *Ilex sugerokii* | 1 |
| 73 | *Illicium philippinense* | 4 |
| 74 | *Juniperus rigida* | 2 |
| 75 | *Kong tuot prei* | 1 |
| 76 | *Krotum prei* | 1 |
| 77 | *Larix cajanderi* | 3 |
| 78 | *Larix gmelinii* | 7 |
| 79 | *Larix kaempferi* | 47 |
| 80 | *Ligustrum japonicum* | 8 |
| 81 | *Lindera umbellata* | 2 |
| 82 | *Litsea acuminata* | 1 |
| 83 | *Litsea noronhae* | 1 |
| 84 | *Litsea* sp. | 1 |
| 85 | *Lyonia ovalifolia* | 5 |
| 86 | *Machilus japonica* | 1 |
| 87 | *Machilus thunbergii* | 2 |
| 88 | *Magnolia kobus* | 1 |
| 89 | *Magnolia obovata* | 12 |
| 90 | *Magnolia salicifolia* | 1 |
| 91 | *Mallotus echinatus* | 2 |
| 92 | *Mallotus japonicus* | 1 |

| ID | Species name | Number  of trees |
| --- | --- | --- |
| 93 | *Meliosma rigida* | 2 |
| 94 | *Milletia sericea* | 3 |
| 95 | *Myristica* sp. | 1 |
| 96 | *Myrsine seguinii* | 9 |
| 97 | *Neoscortechinia kingii* | 2 |
| 98 | *Ochanostachys amentacea* | 2 |
| 99 | *Ochanostachys* sp. | 1 |
| 100 | *Ostodes macrophylla* | 1 |
| 101 | *Oxymitra grandiflora* | 1 |
| 102 | *Phaeh* | 3 |
| 103 | *Phdiek* (Anisoptera) | 1 |
| 104 | *Phellodendron amurense* | 1 |
| 105 | *Picea glehnii* | 11 |
| 106 | *Picea jezoensis* | 10 |
| 107 | *Pieris japonica* | 2 |
| 108 | *Pinus densiflora* | 24 |
| 109 | *Pinus thunbergii* | 1 |
| 110 | *Polaquem dasyphyllum* | 1 |
| 111 | *Polyalthia glauca* | 3 |
| 112 | *Polyalthia rumphii* | 1 |
| 113 | *Pometia tomentosa* | 1 |
| 114 | *Pring bai* (Myrtaceae ?) | 1 |
| 115 | *Pro ang surak thom* (Calophullum ?*)* | 1 |
| 116 | *Prodat phnom* | 1 |
| 117 | *Propro* | 1 |
| 118 | *Prunus grayana* | 2 |
| 119 | *Prunus leveilleana* | 1 |
| 120 | *Prunus maximowiczii* | 1 |
| 121 | *Quercus gilva* | 21 |
| 122 | *Quercus glauca* | 18 |
| 123 | *Quercus mongolica* | 16 |
| 124 | *Quercus phillyraeoides* | 8 |
| 125 | *Quercus salicina* | 19 |
| 126 | *Quercus serrata* | 14 |
| 127 | *Quercus sessilifolia* | 2 |
| 128 | *Quercus* spp. | 2 |
| 129 | *Quercus variabilis* | 7 |
| 130 | *Rhododendron macrosepalum* | 2 |
| 131 | *Rong* (Garcinia) | 5 |
| 132 | *San bok unkrong* (Beilschmiedia ?) | 11 |
| 133 | *Sanbol lowain* (Cinnamomum) | 1 |
| 134 | *Santiria operculata* | 1 |
| 135 | *Santiria tomentosa* | 2 |
| 136 | *Schefflera heptaphylla* | 2 |
| 137 | *Shorea laevis* | 2 |
| 138 | *Shorea leprosula* | 1 |

| ID | Species name | Number  of trees |
| --- | --- | --- |
| 139 | *Shorea ovalis* | 1 |
| 140 | *Sindora* sp. | 1 |
| 141 | *Slorat* (Lithocarpus) | 2 |
| 142 | *Sorbus alnifolia* | 7 |
| 143 | *Sorbus commixta* | 2 |
| 144 | *Sou sei mon chuwa* (Xerospermum) | 2 |
| 145 | *Sterculia rubiginosa* | 1 |
| 146 | *Strombosia rotundifolia* | 1 |
| 147 | *Strombosia sp.* | 2 |
| 148 | *Styrax japonicus* | 2 |
| 149 | *Symplocos lancifolia* | 1 |
| 150 | *Symplocos sumuntia* | 1 |
| 151 | *Tilia japonica* | 1 |
| 152 | *Toxicodendron trichocarpum* | 4 |
| 153 | *Troyung* | 4 |
| 154 | Unidentified sp. | 1 |
| 155 | Unknown | 23 |
| 156 | *Vaccinium bracteatum* | 6 |
| 157 | *Vaccinium oldhamii* | 3 |
| 158 | *Viburnum dilatatum* | 2 |
| 159 | *Viburnum erosum* | 1 |
| 160 | *Xanthophyllum heteropleurum* | 2 |
| 161 | *Yuttao* | 1 |

**S4. Explanation of the datafile, “AllData.csv”**

The datafile was prepared using BAAD data^36^. Abbreviations are as follows.

Climate Climatic zone where the data was collected

Site Location where the data were collected

FunctType Plant functional type; evergreen-gymnosperm (EG), deciduous-gymnosperm (DG), evergreen-angiosperm (EA) and deciduous-angiosperm (DA). These types are abbreviated in the main text as EC, DC, EB and DB, respectively.

Sp Species names, or common names when species names were unknown

TreeID ID of trees in the original dataset

H.m Tree height [m]

Hcb.m Crown base height [m]

DBH.cm Stem diameter at breast height [cm], including bark

Dcb.cm Stem diameter at the crown base height, or the height of the stem location just below the lowest living branch [cm], including bark

BreastH.m Definition of breast height. Generally 1.3 m above ground, but there are exceptions.

Abh.m2 Stem cross-sectional area at breast height [m*^2^*], including bark

Acb.m2 Stem cross-sectional area at the crown base height [m*^2^*], including bark

WL.kg Leaf dry mass per tree [kg]

LA.m2 Leaf area per tree [m^2^]

Owner Owner of the dataset

Source Data source
